# Real-time assessment of high-intensity focused ultrasound heating and cavitation with hybrid optoacoustic ultrasound imaging

**DOI:** 10.1016/j.pacs.2023.100508

**Published:** 2023-05-10

**Authors:** Çağla Özsoy, Berkan Lafci, Michael Reiss, Xosé Luís Deán-Ben, Daniel Razansky

**Affiliations:** a Institute for Biomedical Engineering and Institute of Pharmacology and Toxicology, Faculty of Medicine, University of Zurich, Switzerland; b Institute for Biomedical Engineering, Department of Information Technology and Electrical Engineering, ETH Zurich, Switzerland

**Keywords:** Optoacoustic imaging, Photoacoustic imaging, Ultrasound imaging, High-intensity focused ultrasound treatment monitoring

## Abstract

High-intensity focused ultrasound (HIFU) enables localized ablation of biological tissues by capitalizing on the synergistic effects of heating and cavitation. Monitoring of those effects is essential for improving the efficacy and safety of HIFU interventions. Herein, we suggest a hybrid optoacoustic-ultrasound (OPUS) approach for real-time assessment of heating and cavitation processes while providing an essential anatomical reference for accurate localization of the HIFU-induced lesion. Both effects could clearly be observed by exploiting the temperature dependence of optoacoustic (OA) signals and the strong contrast of gas bubbles in pulse-echo ultrasound (US) images. The differences in temperature increase and its rate, as recorded with a thermal camera for different HIFU pressures, evinced the onset of cavitation at the expected pressure threshold. The estimated temperatures based on OA signal variations were also within 10–20 % agreement with the camera readings for temperatures below the coagulation threshold (∼50 °C). Experiments performed in excised tissues as well as in a *post-mortem* mouse demonstrate that both heating and cavitation effects can be effectively visualized and tracked using the OPUS approach. The good sensitivity of the suggested method for HIFU monitoring purposes was manifested by a significant increase in contrast-to-noise ratio within the ablated region by > 10 dB and > 5 dB for the OA and US images, respectively. The hybrid OPUS-based monitoring approach offers the ease of handheld operation thus can readily be implemented in a bedside setting to benefit several types of HIFU treatments used in the clinics.

## 1 Introduction

High intensity focused ultrasound (HIFU) is widely employed as a non-invasive therapeutic approach for the treatment of benign and cancerous tumors [1], cardiac arrhythmias [2] or blood clots [3] (thrombosis) as well as in new applications including drug and gene delivery [4]. HIFU delivers localized and high intensity (100–10,000 W/cm^2^) ultrasound (US) energy to the target tissue, causing tissue damage through two predominant effects [5], [6]. The first effect corresponds to conversion of US (mechanical) energy to heat via viscous absorption. The rapid temperature rise during HIFU exposure, typically exceeding 50 °C, leads to heat-driven irreversible cell damage including coagulative necrosis, protein denaturation and cell lysis [7], [8]. The second effect is cavitation, known to cause irreversible damage by mechanically disrupting cell membrane permeability and altering the structure of cells [4], [9]. Cavitation is produced in gas nuclei stabilized within the tissue that expand to form a bubble when the frequency-dependent negative (rarefactional) pressure and the mechanical index (MI) exceed a certain threshold [10]. Note that the onset of cavitation further depends on the duration of bursts and other sonication parameters [11], as well as on the presence of microbubbles in the sonicated volume [12]. Additionally, bubbles result in trapping and absorption of US energy via multiple scattering or frequency conversion, which leads to an enhanced (complementary) heating effect [13]. More specifically, enhanced heating occurs due to stable cavitation (volumetric oscillation of existing bubbles following US excitation) or inertial cavitation (sudden burst of expanding bubbles resulting in broadband US emission) [13]. Generally, both heating and cavitation may result in collateral damage to surrounding healthy tissues during HIFU treatments. Thereby, monitoring of HIFU-induced effects is essential for improving the efficacy and safety of these interventions.

Much like for other ablation procedures, HIFU can be monitored by measuring the temperature rise or the cavitation effects at the target tissue. Temperature monitoring has been done with invasive methods e.g. based on thermocouples or fiber-optic temperature sensors [14]. However, noninvasive imaging arguably represents the most convenient approach to quantify the extent of the affected area. HIFU procedures are commonly performed under magnetic resonance imaging (MRI) [9] or US [15] guidance. MRI thermometry provides a good temperature control and monitoring capability [16], but is afflicted by important drawbacks such as low temporal resolution, high operational and equipment costs as well as magnetic compatibility issues. On the other hand, US-based monitoring may offer a more useable and low-cost alternative [17]. This approach mainly relies on detecting hyperechoic regions in the images ascribed to cavitation and other effects [18]. Other methods for cavitation monitoring include harmonic frequency detection [19] and passive acoustic mapping methods [20]. Also, US imaging can provide temperature measurements based on local changes of speed of sound recorded via speckle tracking or other US-based speed of sound measurements [21], [22], [23], [24], [25], [26], [27], [28]. The sensitivity and accuracy of temperature mapping with US imaging are however relatively low due to the complex physical nature of acoustic tissue properties and mechanisms of cavitation [29].

The strong dependence of optoacoustically-induced signals on temperature has also been exploited for the temperature monitoring purposes [30], [31], [32], [33], [34], [35], [36], [37]. Below 50 °C, it has been shown that the generated optoacoustic (OA) signal amplitude linearly scales with the temperature increase. This is mainly attributed to the temperature dependence of the Grüneisen parameter in water [38], [39]. Given this relationship, most OA thermometry methods have been based on quantifying relative temperature changes [40], although absolute temperature measurements have also been showcased [41], [42]. Since protein denaturation and coagulation effects further lead to alteration of optical tissue properties, the linear relationship between OA signal change and temperature increase no longer holds above 50 °C. However, high contrast visualization of the growing thermal lesion is possible for higher temperatures [43]. The relationship between OA signal amplitude and temperature above the coagulation threshold has been calibrated *ex vivo* [44], although this approach may provide inaccurate results in a complex *in vivo* environment. Being based on optical absorption at multiple wavelengths, multi-spectral OA tomography further enables spectroscopic differentiation of coagulated tissues resulting from HIFU or other ablation methods [45], [46], [47]. Vessel disruption [48] and consequent accumulation of deoxygenated blood [49] could also be observed with OA. Thermometry and monitoring during HIFU treatments have been realized with different OA systems and methods [46], [50], [51], [52], [53], [54]. For instance, the thermal dose, an indicator of tissue coagulation, was estimated by combining one-dimensional OA signals and thermocouple recordings [53]. However, 2D or 3D imaging is required to assess potential damage in surrounding tissues. Several studies assessed the extent of the heat-affected area with OA images acquired using linear transducer arrays for different HIFU power levels [55], [56]. In a more recent work, simultaneous real-time OA thermometry and pulse-echo US monitoring has been achieved with a clinical linear-array-based hybrid system [57].

While monitoring of HIFU-induced thermal effects has been extensively investigated, the accompanying cavitation effects were not observed or discussed. In this work, we characterized the cavitation onset with phantom experiments, devised a tailored pulse-echo transmission-reception scheme and developed a combined OPUS imaging approach for HIFU monitoring based on a dedicated multi-segment array. With this, we achieved high-frame-rate visualization and monitoring of both heating and cavitation processes. The OPUS imaging approach further allowed temperature estimation based on OA signal increase during the HIFU exposure and characterization of tissue displacements stemming from the acoustic radiation force in OA and US images.

## 2 Methods

### 2.1 Optoacoustic-ultrasound (OPUS) imaging system

**Fig. 1:**
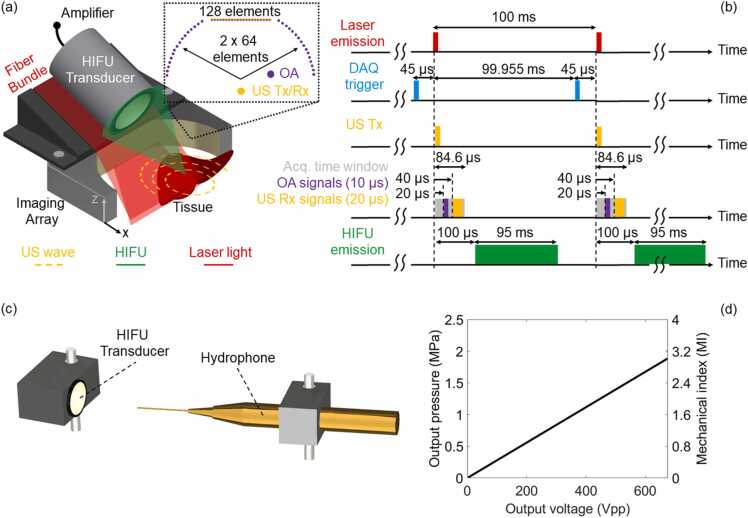
Schematic of the proposed OPUS-based real-time HIFU monitoring. (a) Lay-out of the experimental set-up. Inlet displays the distribution of elements, i.e., 128 elements in the linear and 128 (2 ×64) elements in the two concave segments of the multi-segment array. (b) Timing diagram showing the synchronization between hybrid OPUS imaging sequences and HIFU excitation. DAQ - data acquisition system; OA - optoacoustic; US - ultrasound; HIFU – high-intensity focused ultrasound; Tx - transmission; Rx – reception; Acq. – acquisition. (c) Lay-out of the experimental set-up for the acoustic pressure measurement. (d) Acoustic pressure (MPa) measured at the HIFU focus with a hydrophone and calculated mechanical index (MI) as a function of the peak-to-peak driving voltage (Vpp) of the HIFU transducer.

OPUS imaging was performed with a custom-made multi-segment array (Imasonic SaS, Voray, France) providing optimal performance in both US and OA imaging modes, as described elsewhere [58]. In brief, it consists of one linear (128 elements) and two concave (2 ×64 elements) segments incorporating 256 elements in total and providing a broad tomographic coverage of 170° around the imaged area (Fig. 1a), which is crucial for attaining optimal OA image quality. The elements in the linear and concave segments have an inter-element pitch of 0.25 mm (1.25λ acoustic wavelength) and 0.6 mm (3λ acoustic wavelength), respectively. All elements have ∼70 % detection bandwidth around a central frequency of 7.5 MHz and are shaped to provide cylindrical focusing into the imaged plane. This results in an in-plane resolution of 250 µm and 110 µm for the pulse-echo US and OA modalities, respectively [58]. OA excitation was performed with a short-pulsed optical parametric oscillator (OPO)-based laser (Innolas GmbH, Krailling, Germany) operating at 10 Hz pulse repetition frequency and tuned to λ = 720 nm optical wavelength corresponding to the maximum per-pulse energy within the tunable wavelength range. The laser beam was coupled to a custom-made fiber bundle (Ceramoptec GmbH, Bonn, Germany) split into two linear output arms. These were tilted at ∼17° in the elevational direction to efficiently deliver the light beam onto the imaged plane (Supplementary Fig. S1a-c). The parts required to attach the bundle to the array were specifically designed and 3D printed in polylactic acid (PLA). The measured per pulse fluence at the output of the fiber bundle was approximately 12 mJ/cm^2^, i.e., below the laser exposure safety standards for human skin (20 mJ/cm^2^) at near-infrared wavelengths [59]. The signals collected with the array elements were digitized at 24 megasamples per second (MSPS) rate by a custom-designed data acquisition/transmission unit (DAQ, Falkenstein Mikrosysteme GmbH, Taufkirchen, Germany). A personal computer (PC) was used to control the laser and store the data transmitted from the DAQ via Ethernet. All OPUS imaging sessions were performed with live preview guidance for optimal positioning of the samples. The raw data were saved for further off-line processing.

### 2.2 Acquisition sequence

Synchronization between OA signal acquisition and transmission (Tx) and reception (Rx) of US is schematically depicted in Fig. 1b. The Q-switch output of the laser served as the master trigger source. It was used as an external trigger signal for a function generator (DG1022A, Rigol Technologies Inc., Beijing, China) creating a pulse delayed by 99.955 ms, i.e., 45 μs before emission of the next laser pulse. Due to the internal delay, the data acquisition (DAQ) system was triggered with the same signal. A single short (<1 μs) US Tx bipolar pulse (38 Vpp, 7.5 MHz) was emitted 45 μs after the DAQ trigger (simultaneously with the laser emission) by all 128 elements of the linear array segment. The short duration of US Tx emission ensured that no overlap occurs with the recorded OA signals. Note that it takes ∼20 μs and ∼40 μs to traverse the ∼30 mm distance between the array and tissue surface for the OA and pulse-echo US signals, respectively. Assuming speed of sound of 1500 m/s, the corresponding effective durations of the OA and US signals needed to cover an axial field of view (FOV) of 1.5 cm are 10 μs and 20 μs, respectively. The number of recorded samples (2030) corresponds to an acquisition time window of ∼84.6 μs when sampling at 24 MSPS, which covers the time required to acquire both OA signals and US echoes reflected from the imaged FOV. With the suggested synchronized OA acquisition and US transmission-reception scheme, the signals required for rendering an image are collected in less than 100 µs. Thereby, the frame rate can be readily accelerated if appropriate laser and data acquisition electronics are available. We additionally used a second function generator (DG1022A, Rigol Technologies Inc., Beijing, China) to further delay the HIFU emission by 100 μs so that it does not interfere with the acquired signals.

### 2.3 Image reconstruction

US image reconstruction was performed with a standard delay-and-sum algorithm [60] in a FOV of 30 × 30 mm^2^ with a pixel size of 60 µm. The reconstructed US images were then converted into the decibel (dB) scale. OA images were reconstructed for the same FOV using a back-projection algorithm [61]. The OA and US were normalized to the maximum value of the entire recorded image sequence to better compare the images and to visualize the thermal and cavitation effects. OA signals have broadband frequency content encoding information about physical structures at different scales. In general, high frequency signals represent the fine structures such as small capillaries whereas low frequency components represent the bulk tissue, such as different organs [62]. Prior to reconstruction, the OA signals were band-pass filtered between 0.1 MHz and 8 MHz to better visualize the tissue background and large organs. The speed of sound values (assumed uniform) for US and OA image reconstruction were 1490 and 1470 m/s for the *post-mortem* mouse and the *ex vivo* liver tissue specimens, respectively.

### 2.4 High-intensity focused ultrasound (HIFU) system

HIFU waves were generated with a single-element 36 mm diameter spherically focused transducer having 55 mm focal length and 0.5 MHz central frequency (TQN36–05 C/55, Siansonic Technology Co., Ltd., Beijing, China). The transducer was positioned on top of the fiber bundle holder and oriented at ∼50 ° angle with respect to the elevation direction (Fig. 1a). This ensures HIFU delivery to a superficial region located within the plane covered by OPUS imaging. A power amplifier (ENI 2100 L RF) providing 55 dB amplification in the 10 kHz-12 MHz range was connected to the HIFU transducer. A sinusoidal burst with 0.5 MHz frequency and 47,500 pulses (95 ms), provided by a waveform generator (DG1022A, Rigol Technologies Inc., Beijing, China), was input into the power amplifier. The voltage was adjusted in each experiment.

### 2.5 Acoustic pressure measurements and mechanical index

The US pressure (MPa) at the focus of the HIFU transducer as a function of the peak-to-peak voltage (Vpp) was measured by a calibrated 75 µm diameter hydrophone (Precision Acoustics, UK) facing the focus of the HIFU transducer (Fig. 1c-d and Supplementary Fig. S2). The MI, i.e., peak negative pressure divided by the square root of the center frequency of the acoustic wave, serves as an indicator for the non-thermal effects produced during HIFU exposure that has been shown to correspond to the cavitation probability [63]. FDA regulations for systems and transducers used for diagnostic US define a mechanical index of 1.9 as the safety limit for US exposure due to cavitation events occurring above this threshold [10]. The graph in Fig. 1d thus indicates the peak-to-peak voltage for which cavitation is produced, which enabled validating its occurrence.

### 2.6 Phantom characterization experiments

**Fig. 2:**
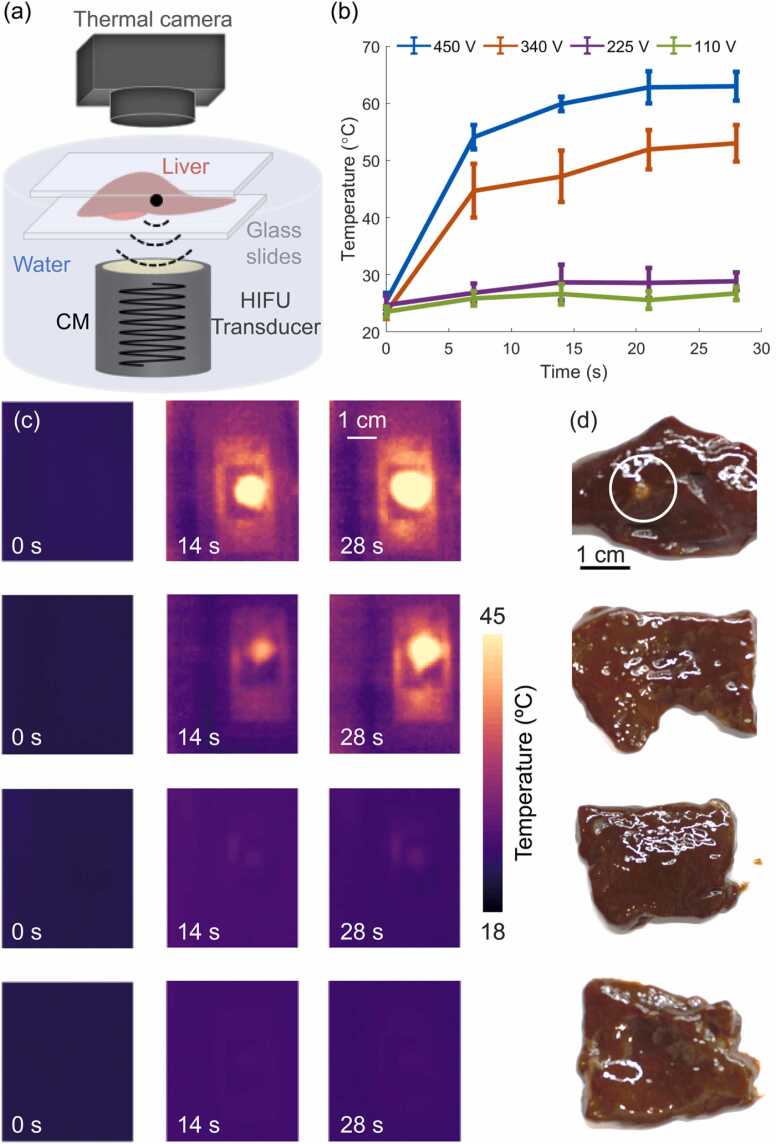
Thermal characterization of HIFU ablation in *ex vivo* porcine liver specimens in a continuous mode (CM). (a) Lay-out of the temperature measurement and HIFU ablation set-up. (b) Temperature profiles recorded by the thermal camera located above the liver sample close to the HIFU focus for peak-to-peak driving voltages of 450 V (blue profile), 340 V (orange profile), 225 V (purple profile) and 110 V (green profile), respectively. Error bars represent the standard deviation of the temperature at a region around the HIFU focus. (c) Images acquired with the thermal camera for peak-to-peak voltages of 450 V (first row), 340 V (second row), 225 V (third row) and 110 V (fourth row). (d) Photographs of the ablated liver specimens for peak-to-peak voltages of 450 V, 340 V, 225 V and 110 V, respectively.

**Fig. 3:**
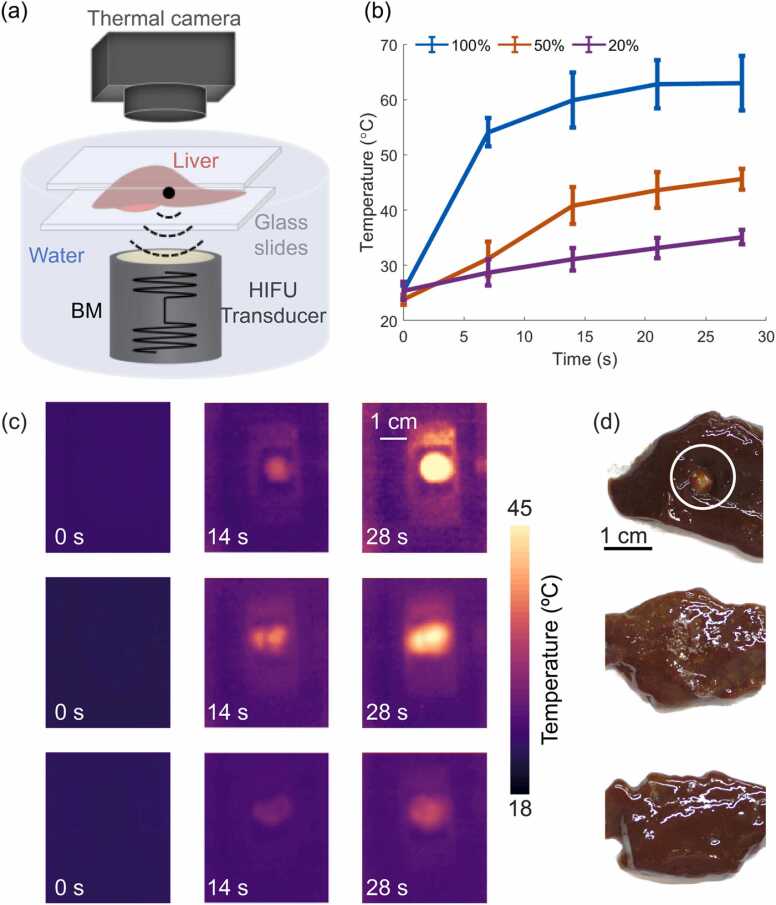
Thermal characterization of HIFU ablation for *ex vivo* porcine liver specimens in burst mode (BM). (a) Lay-out of the temperature measurement and HIFU ablation set-up. (b) Temperature profiles recorded by the thermal camera located above the liver sample close to the HIFU focus for peak-to-peak driving voltage of 450 V with different duty cycles, namely 100 % (blue profile), 50 % (orange profile) and 20 % (purple profile), respectively. Error bars represent the standard deviation of the temperature at a region around the HIFU focus. (c) Images acquired with the thermal camera for peak-to-peak voltage of 450 V and duty cycles 100 % (first row), 50 % (second row) and 20 % (third row), respectively. (d) Photographs of the ablated liver specimens for peak-to-peak voltage of 450 V, duty cycles of 100 %, 50 % and 20 %, respectively.

The HIFU ablation performance was first tested by measuring the temperature and examining the generated lesion in *ex vivo* porcine liver tissues using different driving voltages in continuous mode (110 V, 225 V, 340 V and 450 V, Fig. 2) and different duty cycles in burst mode (450 V, 20 %, 50 % and 100 %, Fig. 3). HIFU parameters were selected based on the measured acoustic pressure and calculated MI. The driving voltages were chosen to be around 430 V, corresponding to the cavitation threshold of ∼1.3 MPa at 0.5 MHz. The effects of varying duty cycle on HIFU-induced treatments have previously been investigated [64], so that the efficacy of the ablation treatment when different duty cycles are employed can be controlled. It has been shown that higher duty cycles lead to increased cavitation activity that enhances the lesion size [65]. Here, both high (100 % and 50 %) and low (20 %) duty cycles regimes were tested to assess the efficacy of the HIFU treatment protocol. Furthermore, this type of soft tissue is known to have relatively high US attenuation [66], thus facilitating HIFU heating. A simplified schematic representation of the temperature monitoring set-ups is depicted in Figs. 2a and 3a. The actual experimental set-up of the thermal camera measurement during HIFU ablation can be found in Supplementary Fig. S3. Tissue specimens were fixed between two ∼150 µm thick glass slides, which can be considered to be acoustically transparent for the given HIFU frequency (Figs. 2a and 3a) thus averting any tissue displacement caused by the acoustic forces. Temperature monitoring during HIFU ablation was done by a handheld thermal imaging camera (UTi-85A, [−10 °C 400 °C], UNI-Trend Technology Co., Ltd., China) at different time points before (0 s) and during (until 28 s) HIFU exposure. For this, the HIFU transducer was placed vertically below the *ex vivo* liver specimen (Figs. 2a and 3a) and immersed in a water tank. The water was degassed prior to the experiments to avoid bubble formation within the aqueous medium (DEGASI® High Flow Degasser, Biotech AB, Onsala, Sweden). The thermal camera was placed on top of the sample.

### 2.7 OPUS monitoring of HIFU ablation in excised tissues

Excised *ex vivo* porcine liver tissue specimens were also used in a second experiment to assess the performance of OPUS imaging for monitoring HIFU ablation. This type of tissue has relatively high optical absorption [67], which facilitates delineation of ablation lesions in OA images. The set-up described in Section 2.1 and depicted in Fig. 1a was also used in these experiments. A photograph of the experimental set-up of the OPUS visualization of HIFU lesion progression in a fixed *ex vivo* liver specimen can be found in Supplementary Fig. S4. Much like in the characterization experiments, the water was degassed prior to the experiment. During the first OPUS monitoring session, the *ex vivo* porcine liver specimens were held between two thin glass slides (∼150 µm) to avoid tissue displacement caused by acoustic forces. OPUS imaging was performed for 30 s during HIFU exposure (which started and ended with the OPUS data acquisition) at peak-to-peak voltage values of 500 V, 340 V and 170 V in continuous mode. The temperature was also measured with the thermal camera at the same peak-to-peak voltage values in continuous mode for 30 s, and compared to the temperature estimated using OA signals based on a previously described method [31]. Briefly, the temperature estimation method is based on the temperature dependence of the Grüneisen parameter, which defines the thermoelastic efficiency of the absorbing media by incorporating volumetric thermal expansion, speed of sound and specific heat capacity parameters [33], [68]. The temperature dependence of the Grüneisen parameter for an aqueous solution is approximated via [69].

$$\Gamma \left(T\right)=0.0043+0.0053T,$$

where T is the temperature in °C. Considering that OA signals are proportional to the Grüneisen parameter for given optical absorption coefficient and optical fluence, the temperature increase can be calculated as

$$\Delta T=\frac{(0.0043+0.0053T_{0})\Delta p}{0.0053p_{0}}.$$

This temperature estimation method is valid below the coagulation threshold (∼50 °C) as beyond this point the Grüneisen parameter and the optical properties of the tissue are expected to change in a non-linear fashion [32], [70].

In a second OPUS monitoring experiment, the *ex vivo* porcine liver sample was held with two needles inside a water tank. This allows displacement due to acoustic forces, thus resembling a more realistic scenario. A sequence of OPUS images was acquired during the HIFU ablation procedure for 90 s, including 10 s before HIFU exposure, 50 s during exposure and 30 s during the cool-down period. The peak-to-peak voltage at the HIFU transducer was set to 560 V.

### 2.8 OPUS monitoring of HIFU ablation in a mouse *post-mortem*

The performance of OPUS imaging for monitoring HIFU ablation was also assessed in an 8-weeks-old female athymic nude-Fox1nu mouse *post-mortem*. The experiment was performed in full accordance with the Swiss Federal Act on Animal Protection and approved by the Cantonal Veterinary Office Zurich. A simplified schematic representation of the experimental setup is depicted in Fig. 6a. HIFU ablation was targeted at the liver region (left side). Much like in the second excised tissue experiment described in Section 2.1, a time-lapse sequence of OPUS images was acquired during the HIFU ablation procedure for 180 s, including 30 s prior to HIFU exposure, 60 s during HIFU exposure and 90 s during the cool-down period. The peak-to-peak voltage at the HIFU transducer was set to 560 V. Following the OPUS imaging experiment, the ablated region was prepared for cryo-sectioning to verify the HIFU ablation. For this, the sample was placed in a plastic tube, embedded in the optimal cutting temperature compound (Tissue-Tek® O.C.T., Sakura Finetek USA, Inc.) and frozen in a − 80 °C freezer. The frozen sample was then sectioned with the slice thickness adjusted to 100 µm.

## 3 Results

The temperature changes induced in the *ex vivo* porcine liver tissue during HIFU exposure were first quantified. This was done by considering the thermal maps acquired with a handheld thermal imaging camera as described in Section 2.6. Two HIFU operation modes (continuous and burst) were considered with parameters defined in Section 2.6. Thermal maps obtained at different time points, peak-to-peak voltages (continuous mode), and peak-to-peak voltages and duty cycles (burst mode) are depicted in Figs. 2c and 3c, respectively. Photographs of the ablated liver specimens corresponding to each row of continuous (Fig. 2d) and burst (Fig. 3d) modes of HIFU operation are also shown. As expected, a higher driving voltage in the HIFU transducer led to a higher temperature increase in the ablated region (Fig. 2b). More specifically, the temperature increases were ∼30 °C and ∼38 °C for driving voltages of 340 V and 450 V, respectively, while these were only ∼4 °C for 225 V and ∼3 °C for 110 V. The sharpest temperature increase occurred in the first 7 s, which is attributed to the immediate generation of bubbles at the beginning of HIFU exposure. The slopes during the first 7 s were 4.1 °C/sec and 3.0 °C/sec for 450 V and 340 V, respectively, being proportional to the driving voltage in this range. On the other hand, for 225 V the slope during the first 7 s was 0.4 °C/sec. This significant differences between the temperatures achieved with 225 V and 340 V driving voltages (Fig. 2b) and the corresponding rates of change are ascribed to the nonlinear tissue-specific cavitation threshold behavior [13], which contributes to accelerating the heating process. This is in agreement with the MI for which the onset of cavitation is expected (Fig. 1d). Generally, tissues undergo ablation when the temperature reaches a coagulation threshold of ∼50 °C [7] with the cell death exponentially increasing with temperature in the 40–55 °C temperature range [71]. The time required for tissue ablation in this range has thus been heuristically established as a function of temperature [72].

The results obtained further confirmed that the temperature rise monotonically increases with the duty cycle (Fig. 3b and c). More specifically, for 450 V driving voltage the temperature increases were ∼10 °C, ∼22 °C and ∼38 °C for duty cycles of 20 %, 50 % and 100 %, respectively. Note that slight differences are expected due to the complex (heterogeneous) structure of the liver tissue. The slope of the observed temperature rise achieved with sinusoidal bursts corresponding to duty cycles of 20 %, 50 % and 100 % at 450 V peak-to-peak voltage (Fig. 3b) during the first 7 s were 0.4 °C/s, 1.1 °C/s and 4.1 °C/sec, respectively. The lack of linear relationship between duty cycle and temperature rise is attributed to thermal diffusion as well as stabilization of dissolved gas within the tissue during the 20 % and 50 % duty cycle exposures. It is also important to note that lower acoustic energy was delivered to the tissue for 450 V peak-to-peak voltage at 20 % duty cycle (Fig. 3b) as compared to 225 V peak-to-peak voltage at 100 % duty cycle (continuous mode, Fig. 2b). Yet, a higher temperature increase (10 °C at 20 % duty cycle versus 4 °C in continuous mode) was observed, which is consistent with the fact that cavitation is induced in the former case leading to an enhanced temperature rise. In our experiments, larger lesions were observed (Fig. 2d, first row and Fig. 3d, first row) when the HIFU transducer was excited with a high peak-to-peak voltage of 450 V, resulting in a temperature rise up to 60 °C (Fig. 2c, first row and Fig. 3c, first row).

**Fig. 4:**
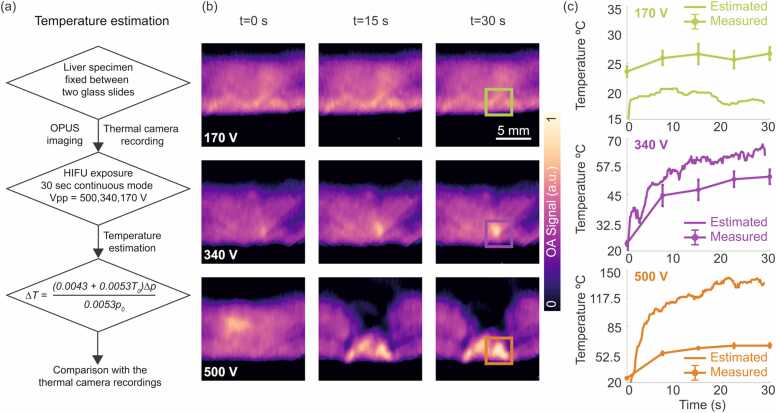
Temperature estimation method and OA visualization of HIFU lesion progression in a fixed *ex vivo* liver specimen. (a) Flow diagram of the temperature estimation method using OA signals obtained from the fixed liver specimen. (b) OA images at the beginning (t = 0 s), middle (t = 15 s) and end (t = 30 s) of HIFU exposure for peak-to-peak voltage values of 170 V (first row), 340 V (middle row) and 500 V (third row) in continuous mode. Scalebar – 5 mm. (c) Temperature estimated from the OA signal variations (solid green, solid purple and solid orange curves) versus temperature measured using the thermal camera (dotted solid green, dotted solid purple and dotted solid orange curves) for three driving voltages. The regions of interest considered for the estimated temperatures are marked in green, purple and orange squares in (b).

Changes in OA signal intensity can be used to estimate the temperature increase as described in Section 2.7 and depicted in Fig. 4a. Note that this is only possible for temperatures below the coagulation threshold, as changes in optical properties and subsequent non-linear changes of OA signal intensity with temperature are produced at higher temperatures. Fig. 4b displays the OA images captured at three instants (t = 0 s, 15 s and 30 s) during the HIFU ablation procedure. A progressive increase in OA signal intensity as the temperature increases is visible for 340 V (Fig. 4b, second row) and 500 V (Fig. 4b, third row) HIFU driving voltages, whereas the OA signal intensity remained fairly stable for 170 V (Fig. 4b, first row) HIFU driving voltage. More specifically, the normalized OA intensities in the regions of interest (ROIs) marked with green, purple and orange squares in Fig. 4b increased 72 %, 23 % and 5 % from their baselines during the first 7 s at 500 V, 340 V and 170 V, respectively. Similar to the thermal camera measurements, cavitation produces an enhanced increase of temperature, arguably occurring at driving voltages beyond 340 V. Note that the set-up configuration and orientation of the HIFU transducer relative to the tissue is different than in the thermal camera experiments. The temperature increase obtained from the thermal camera readings during the first 7 s were 113 %, 91 % and 9 % at 450 V, 340 V and 225 V, respectively.

The temperature increase ΔT was estimated (Fig. 4c) as described in Section 2.7 (flow diagram in Fig. 4a), where the measured initial temperature was 20 °C. The relative OA signal increments were calculated considering a baseline OA image acquired prior to the ablation procedure. Specifically, the ROIs marked with green, purple and orange squares in Fig. 4b were considered. The temperatures measured with the thermal camera are also shown in Fig. 4c for three driving voltages. The OA-estimated temperature values with peak-to-peak driving voltages of 170 V and 340 V provide an overall good estimation as verified with the thermal camera measurements. More specifically, the OA-estimated temperature values with peak-to-peak driving voltage of 170 V underestimate the temperature by ∼20 % at 10 s and at 30 s while 340 V overestimate the temperature by ∼12 % at 10 s and ∼19 % at 30 s compared to the thermal camera measurements. These uncertainties are expected considering that the temperature dependence of the Grüneisen parameter in actual heterogenous biological tissues is not exactly the same as that in water. Furthermore, cavitation may also change the Grüneisen parameter of the tissue, which also affects the OA signal and causes errors in the temperature estimations. The large discrepancy between the estimated and measured temperature at peak-to-peak driving voltage of 500 V was attributed to the non-linear changes in the Grüneisen parameter and tissue optical properties above the coagulation threshold of ∼50 °C.

**Fig. 5:**
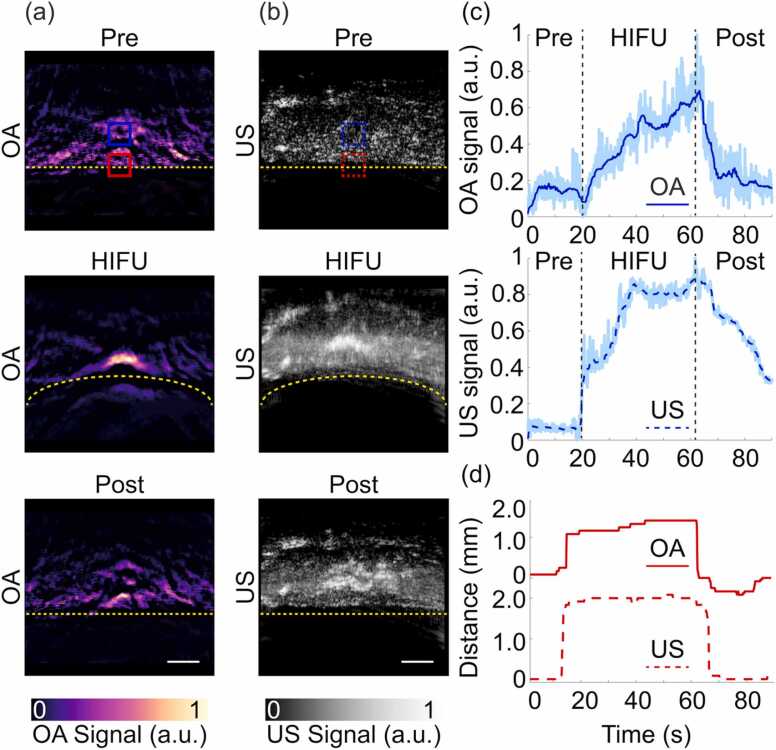
OPUS visualization of HIFU lesion progression in a not fixed *ex vivo* liver specimen. (a) OA images before (Pre), during (HIFU) and after (Post) HIFU ablation. Scalebar – 3 mm. (b) Corresponding US images before (Pre), during (HIFU) and after (Post) HIFU ablation. Scalebar – 3 mm. Yellow dashed lines delineate the tissue boundary. (c) Time-lapse OA and US readings in the region-of-interest (ROI) indicated with blue squares in (a) and (b). Dark blue curves show the signals after applying a moving average filter with a window size of 30 while the raw (unfiltered) signals are shown in the background. The vertical black dashed lines indicate time points before (Pre), during (HIFU) and after (Post) HIFU exposure. (d) Tissue displacements calculated using the surface OA and US signal intensities in the blue ROIs (a) and (b).

The feasibility of HIFU ablation monitoring with OPUS was then evaluated with an *ex vivo* porcine liver specimen as described in Section 2.7. OPUS images were acquired during the entire procedure. The acquired sequence of images is displayed in a movie available in the online version of the journal. The reconstructed OA and US images were normalized separately to the maximum value of each sequence. Three representative examples of images taken before, during and after HIFU exposure are shown in Fig. 5a and b, respectively. A clear signal increase at the HIFU focus was observed with both modalities. The signal levels decreased after HIFU exposure, although remaining above the baseline values at the beginning of the experiment. Additionally, tissue displacement resulting from the acoustic radiation force [73] is clearly visible, particularly in the US images. The tissue boundary, whose original position (0 mm) is indicated with a yellow dashed line in Fig. 5a, was shifted (curled) up to 2 mm during HIFU exposure but returned to the initial position (0 mm) after ablation was completed. A more detailed analysis of the signal profiles at the HIFU focus (Fig. 5c) facilitates assessment of both the heating and cavitation effects. These were assessed by calculating the maximum values across the selected region (blue square in Fig. 5a and b) over the course of 90 s to avoid the influence of tissue displacement. The OA signal is shown to increase with time, which indicates that it is primarily associated with the temperature increase. Note, however, that OA signals are also affected by cavitation as well as by changes in tissue optical absorption due to coagulation. On the other hand, the US signal experiences a sharp increase in the initial ablation phase, arguably implying that cavitation is generated almost immediately [74]. We ascribe the subsequent signal rise to an increase in the cavitation activity caused by heating. The US signal eventually reaches a plateau, which appears to indicate that bubble formation saturates, before gradually decreasing after HIFU ablation was terminated. The tissue displacement observed in the OPUS images was quantified by identifying the tissue surface in the OA and US images (Fig. 5d). The initial position was set to 0 mm and approximately 2 mm tissue displacement were observed in both OA and US images. The differences in the profiles are ascribed to changes in speed of sound caused by heating, which affected differently OA and US images as the acoustic waves involved in image reconstruction propagate along different trajectories for the two modalities. Hybrid OPUS imaging then provides a more robust estimation of the tissue displacement. The lesions generated at the HIFU focus had an asymmetric shape, i.e., tadpole shape which was previously observed during HIFU ablation experiments [75]. Additional lesion enlargement can be attributed to scattering of the acoustic energy by the bubbles, which consequently results in more energy being absorbed in the ablation zone [76].

**Fig. 6:**
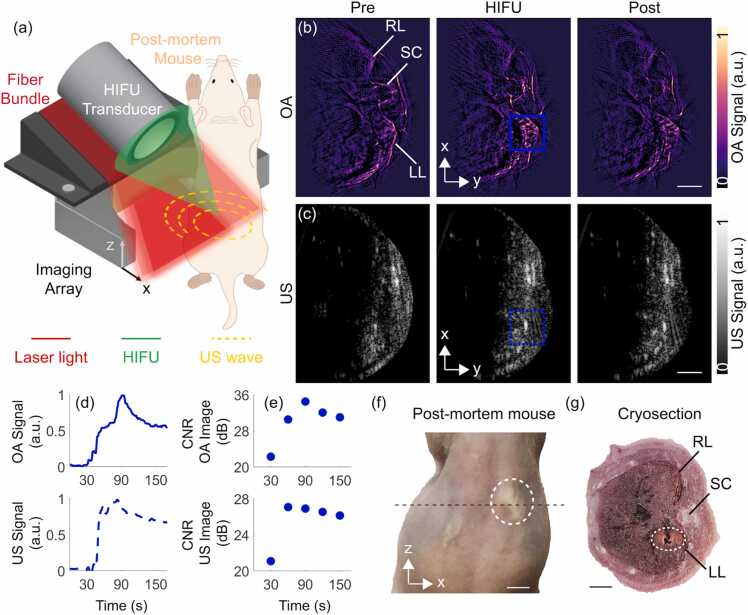
Real—time visualization of HIFU ablation progression in a *post-mortem* mouse with OPUS. (a) Lay-out of the experimental set-up of OPUS monitoring of HIFU ablation in a mouse *post-mortem*. (b) OA images before (Pre), during (HIFU) and after (Post) HIFU ablation. LL – Liver (left), SC – Spinal cord, RL – Liver (right). (c) Corresponding US images before (Pre), during (HIFU) and after HIFU (Post) ablation. The individual OA and US frames were normalized separately to the maximum values of the corresponding time-lapse image sequence. (d) Time-lapse OA (blue solid line) and US (blue dashed line) readings in the region-of-interest (ROI) indicated with blue squares in (b) and (c). (e) Contrast-to-noise ratio (CNR) of OA (top) and US (bottom) images in dB calculated prior (30 s) and during (60 s, 90 s, 120 s, 150 s) HIFU ablation. (f) Photograph of the ablated *post-mortem* mouse. The ablated region is marked with a white dashed circle. The OA and US images correspond to the cross-section marked with the black dashed line. (g) Cryosection of the liver region of the *post-mortem* mouse after the HIFU ablation procedure (cross-section corresponds to the black dashed line in panel e). The ablated region is marked with a white dashed circle. Scalebar corresponds to 5 mm in panel f and 3 mm in all other panels.

Finally, we tested the capabilities of OPUS monitoring of HIFU-induced heating and cavitation in a more heterogeneous sample better mimicking *in vivo* condition. For this, we performed HIFU ablation targeting the liver region of a mouse *post-mortem* (Fig. 6). The signals were acquired for 180 s including 30 s baseline, 60 s during HIFU exposure and 90 s during the cool-down period. Both OA and US images enable visualizing main anatomical structures in the mouse such as the liver or the spinal cord (Fig. 6b and c). The gradual effects and structural changes of HIFU ablation in the *post-mortem* mouse can be best perceived in the full-length movie available in the online version of the journal. Three representative OA and US images taken before, during and after HIFU exposure are displayed in Fig. 6b and c, respectively. A signal increase in the ablated region was observed in both modalities (blue squares in Fig. 6b and c), arguably corresponding to heating and cavitation. For a more quantitative assessment, time trace curves were extracted from the OA and US images at the HIFU exposed region (Fig. 6d). Similar to the OA time trace for the *ex vivo* liver specimen (Fig. 5c), the OA signal in the *post-mortem* mouse increased with time as a result of the temperature increase, acoustic cavitation and changes in tissue optical properties due to coagulation (Fig. 6d). Specifically, a slight increase in OA signal intensity was first observed, arguably due to a temperature rise.

The subsequent change in slope of the OA signal intensity curve is ascribed to the onset of cavitation and the associated accelerated heating. At a later time point, another change in slope was detected in the OA signal intensity curve, arguably corresponding to coagulation. The pulse-echo US signal also underwent a sharp increase matching the change in slope of the OA signal variations, further substantiating the appearance of cavitation effects. The enhanced contrast before (30 s) and after ablation (60 s, 90 s, 120 s, 150 s) in the ablated region (blue squares in 6b and 6c) was quantified by calculating the contrast-to-noise ratio (CNR), i.e., the difference of signal values in ablated and background regions normalized to the standard deviation of the noise, shown in Fig. 6e. These CNR graphs serve to verify that OA and US provide sufficient contrast for visualizing combined heating and cavitation effects. Distortion of anatomical structures in the images was also observed, which is ascribed to a combination of acoustic radiation force and local changes in speed of sound due to heating. Coagulation associated to HIFU ablation was visible in the photograph taken from the back of the mouse after the experiment (Fig. 6f, white dashed circle). The presence of an ablated region was further confirmed in the cryo-section of a region approximately matching the imaged cross-section (Fig. 6g, white dashed circle). Slight displacements are attributed to the compression of the sample to fit in the sample holder used to prepare the mouse for the cryoslicer.

## 4 Discussion

OPUS-based guidance with the multi-segment array was shown to provide an efficient means for assessing the effects of HIFU exposure. On the one hand, the amplitude of OA signals is known to provide high sensitivity to temperature changes (∼2.7 % signal change per K in living mammalian tissues [31]), thus OA image changes are mainly associated to thermal effects. On the other hand, bubble formation results in enhanced US back-scattering arguably representing the main cause behind the hyperechoic regions observed in the US images [77]. It is yet important to recognize that heating and cavitation generally lead to signal alterations in both modalities. For example, the presence of bubbles is known to result in enhanced heating and corresponding increase in the amplitude of the generated OA signals [78]. This was validated with the experiments performed with *ex vivo* porcine liver tissues measured with a thermal camera. Also, OA signals are further affected by light absorption changes resulting from tissue coagulation [32]. Adverse effects in tissues such as microvascular injuries may result in US hyperechoic formations [18]. Tissue damage and the presence of gas-filled cavities also lead to attenuation and distortion of US waves and appearance of artifactual features in the images. Overall, accurate modelling of these and other effects is very challenging, thus hampering absolute quantification of heating and cavitation effects.

OA has been shown to provide excellent sensitivity to temperature changes and can additionally sense the effects of coagulation, i.e., optical absorption changes, microvascular alterations and accumulation of blood. Hence, OA imaging has recently been suggested as a powerful method for monitoring a range of other tissue ablation techniques. In particular, real-time OA visualization of the effects induced by radiofrequency ablation [79], [80], [81], cryoablation [82] or laser ablation [44], [83] has been achieved. These ablation techniques generally do not involve acoustic cavitation events, although vaporization may be produced at high temperatures [7]. Tissue destruction can also result in alteration of mechanical properties that are readily visible in the US images, which can further reveal the presence of liquefied regions, tissue disruption or blood flow changes via color Doppler imaging [49]. The hybrid OPUS monitoring approach suggested in this work can thus enhance the capabilities of OA for monitoring a multitude of thermal treatments. Future developments include the integration of a 3D OA imaging system capable of providing more quantitative information. More accurate differentiation between heating and cavitation effects can be achieved if absorption and fluence changes are compensated for with more accurate models accounting for optical and acoustic tissue properties [44], [84], [85], [86], [87]. Indeed, significant efforts have been directed towards the development of OA thermometry methods [36], [44]. However, most of these methods are only valid for temperatures below 50 °C, where the differential OA signal intensity linearly increases with temperature [36], [56]. OA thermometry above the coagulation threshold has also been suggested based on measurements of canine blood absorbance *ex vivo*, although the *in vivo* results varied significantly depending on *ex vivo* calibrations. Furthermore, most studies focused so far on OA monitoring of radiofrequency- and laser-based ablation treatments whereas only a few explored HIFU-based ablation treatment monitoring with OA [46], [50], [51], [52], [53], [54]. HIFU ablation is fundamentally different from other ablation procedures in that both heating and cavitation are generally produced. Recently, Kim et al. characterized the OA signal changes with temperature in an in vitro feasibility experiment and verified that the HIFU-induced temperature rise can be measured from the OA signals with thermocouples implanted in mice *in vivo* [57]. This study was also the first to explore hybrid OA and US monitoring of HIFU treatments. However, cavitation effects were not observed or discussed, whilst only a specific HIFU intensity and duty cycle (50 W, 50 %) was considered. With our approach, the acquisition of OA and US signals takes less than 100 µs, thus ensuring almost continuous HIFU exposure. The *ex vivo* experiments facilitated a thorough characterization of heating- and cavitation-induced tissue damage with different HIFU exposure sequences. This further enabled establishing the minimal pressure required to reach the cavitation threshold and combined thermal effects. Instead of thermocouple recordings, we used thermal camera measurements that provide information over the region of interest exposed to HIFU energy as well as surrounding areas.

Despite the successful visualization of HIFU effects using the complementary contrast provided by OA and US imaging in *ex vivo* liver tissues and *post-mortem* mice, some limitations remain for an optimal characterization of thermal lesions with OPUS. It has previously been demonstrated that high contrast and unambiguous lesion differentiation can be achieved using a single wavelength [51], [53]. However, for more accurate delineation and characterization of the lesion, the absorption spectra of ablated and non-ablated tissues can be taken into account [46]. Multi-spectral OA imaging has previously been suggested for characterizing tissue ablation procedures [46], [47], [88]. However, it is important to consider that multi-spectral imaging effectively reduces the achievable temporal resolution. On the other hand, while optoacoustically-estimated temperature values below the coagulation threshold (50 °C) were in good agreement with the temperature values measured with the thermal camera, a large discrepancy was observed for temperatures above this threshold. This was attributed to an increase in optical absorption when coagulation is produced. However, despite this inaccuracy, tissue ablation (coagulation) can be detected if the temperature estimated from the OA signals exceeds 50 °C. In this case, a stop criterion for the HIFU therapy can be established by also considering the total exposure time. The temperature-dependent absorption properties and optical fluence attenuation can also potentially be accounted for [44], [84], [85], [86], [87] to enhance the quantification capacity of OPUS imaging of HIFU-induced effects. However, it should be noted that an accurate calibration should be performed to account for varying optical absorption properties in heterogeneous living tissues for fluence compensation methods.

An important factor to consider is the different penetration depth provided by OA versus pulse-echo US. For frequencies below 20–30 MHz, the achievable depth is mainly determined by light attenuation in OA and by acoustic attenuation in US [89]. As a result, multi-modal imaging may not be possible at depths beyond 2–3 cm due to limited light penetration into biological tissues. A possible solution consists of endoscopic light delivery to deeper regions, as was previously implemented with radiofrequency ablation catheters [80], [81], [90], [91]. However, such an approach may not be practical in most HIFU interventions. Note also that the speed of sound changes caused by heating or other acoustic aberration effects may result in distortion in the images reconstructed assuming a uniform speed of sound. This distortion is different for OA and US images as different propagating paths and also different array elements are involved, thus the simultaneously recorded multi-modal images may not be perfectly registered. More advanced algorithms may thus be required for proper co-registration and comparison of the images [92]. Accurate modelling of changes in optical properties and the associated light fluence attenuation in heterogeneous tissues can also potentially enhance the quantification capacity of the suggested approach [84], [85], [86], [87]. OPUS imaging systems are currently being translated into the clinics and commercial systems are already available for this purpose [93], [94]. In the context of monitoring of clinical HIFU treatments, key success factors include real-time imaging capability, high spatial resolution and sufficient contrast to ensure safe and effective lesion assessment. Clinical translation may also be facilitated with less expensive and compact solutions e.g. based on pulsed laser diodes [95], [96].

A proper protocol (workflow) must be defined for using the suggested approach in specific clinical applications. Depending on the target therapeutic intervention, human body area and temperature range, the HIFU intensity, duration and other parameters need to be properly calibrated as the effects (heating, cavitation or acoustic radiation force) can significantly alter the outcome. Generally, the US parameters used in HIFU therapy vary depending on the clinical application and the specific device being used. The OPUS imaging array, light delivery method, and HIFU transducer(s) must then be designed accordingly.

## 5 Conclusion

In this study, the feasibility of real-time monitoring of heating and cavitation effects during HIFU exposure was demonstrated using a hybrid OPUS imaging approach. The experiments were performed in both controlled tissue phantoms as well as a more realistic *post-mortem* mice environment. As expected, temperature elevations were generally proportional to the HIFU pressure (driving voltage of the transducer), while significant nonlinear effects were produced for large driving voltages arguably due to cavitation effects. OA images enabled temperature mapping below the tissue coagulation threshold (∼50 °C), while the temperature was overestimated for higher pressures due to changes in the Grüneisen parameter and tissue optical properties. OA can thus be used to determine when the coagulation threshold is reached. It was further possible to quantify the movement induced by acoustic radiation forces with both OA and pulse-echo US modes. The OPUS images exhibited excellent contrast in the ablated region, which allowed an unambiguous identification of both heating and cavitation processes. While OA signals strongly increase with temperature and the onset of cavitation causes hyperechoic contrast in pulse-echo US, it is important to take into account that both effects contribute to changes in the OA and US images. Future work will then be directed toward disentangling the cavitation and coagulation effects by compensating for the absorption and fluence changes with more accurate models accounting for dynamic optical and acoustic tissue properties. In conclusion, the hybrid OPUS monitoring approach was shown capable of simultaneously visualizing and assessing heating and cavitation effects associated to HIFU treatments in real time. Owing to the ease of handheld operation, OPUS can potentially be implemented in a bedside setting to benefit clinical HIFU interventions.

## Funding

This project has received funding from the 10.13039/501100000781European Research Council (ERC) under grant agreement ERC-CoG-2015-CoG-682379 (D.R) and from 10.13039/501100013850Helmut Horten Stiftung (project deep skin, X.L.D.B).

## CRediT authorship contribution statement

X.L.D.B conceived the study. Ç.Ö., X.L.D.B and B.L. performed the experiments. Ç.Ö. processed the experimental data, performed the calculations, prepared the figures and wrote the original draft. X.L.D.B. and B.L. developed the algorithms. X.L.D.B. and D.R. supervised the study. All authors wrote and edited the manuscript.

## Declaration of Competing Interest

The authors declare that they have no known competing financial interests or personal relationships that could have appeared to influence the work reported in this paper.

## Data Availability

Data will be made available on request.
