# Supplementary material for: Real-time assessment of high-intensity focused ultrasound heating and cavitation with hybrid optoacoustic ultrasound imaging
Source: Photoacoustics. 2023 May 10;31:100508. doi: 10.1016/j.pacs.2023.100508 (PMC10203775; doi:10.1016/j.pacs.2023.100508)
Supplement: Supplementary file 1 — Supplementary material [file mmc1.pdf]

Supplementary Information for

Real-time assessment of high-intensity focused ultrasound heating and cavitation with hybrid optoacoustic ultrasound imaging

Çağla Özsoy[a,b], Berkan Lafci[a,b], Michael Reiss[a,b], Xosé Luís Deán-Ben[a,b,*], Daniel Razansky[a,b,*]

[a]Institute for Biomedical Engineering and Institute of Pharmacology and Toxicology, Faculty of Medicine, University of Zurich, Switzerland

[b]Institute for Biomedical Engineering, Department of Information Technology and Electrical Engineering, ETH Zurich, Switzerland

*Corresponding author: xl.deanben@pharma.uzh.ch (X.L.D.-B.), daniel.razansky@uzh.ch (D.R.)

This file includes:

Supplementary Fig. S1 Light profile at the tissue surface for the OPUS imaging array.

Supplementary Fig. S2 Actual photograph of the calibrated hydrophone.

Supplementary Fig. S3 Experimental setup of the thermal camera measurement during HIFU ablation.

Supplementary Fig. S4 Experimental setup of the OPUS visualization of HIFU lesion progression in a fixed ex vivo liver specimen.

Supplementary Figures:

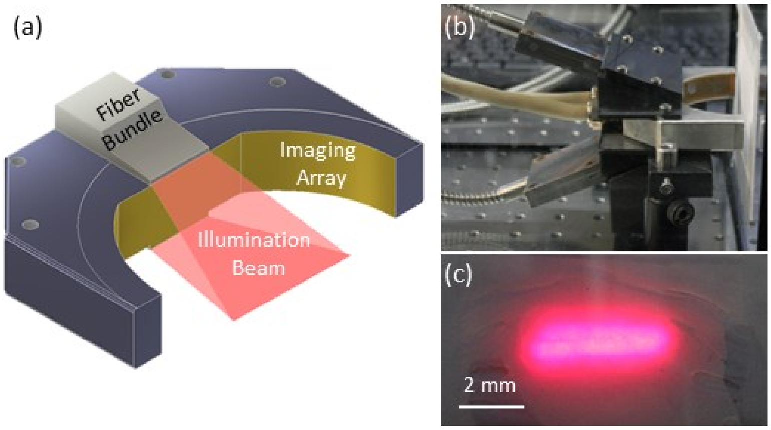

Supplementary Fig. S1. Light profile at the tissue surface for the OPUS imaging array. (a) 3D rendering of the multi-segment hybrid imaging array, including the illumination fiber bundle. (b) Actual photograph of the multi-segment hybrid imaging probe with the attached illumination fiber bundle. (c) Light profile created by the fiber bundle attached to the multi-segment imaging array at the approximate location of the tissue surface.

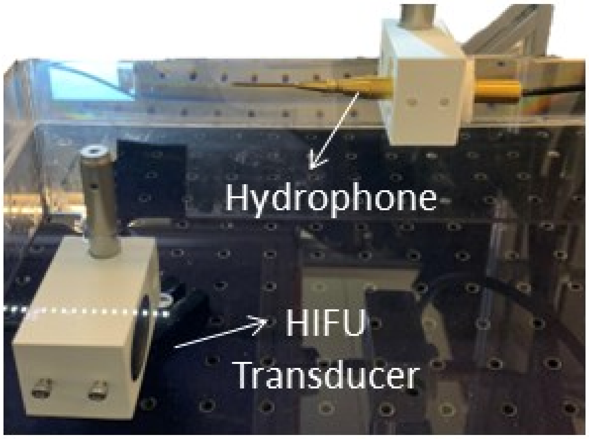

Supplementary Fig. S2. Actual photograph of the calibrated hydrophone. The photograph was taken while scanning towards the HIFU transducer focus to measure the acoustic pressure (MPa) as a function of the peak-to-peak voltage (Vpp).

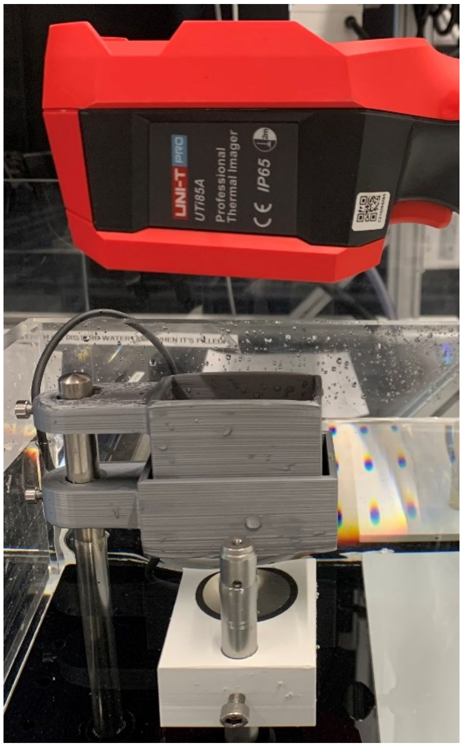

Supplementary Fig. S3. Experimental setup of the thermal camera measurements during HIFU ablation.

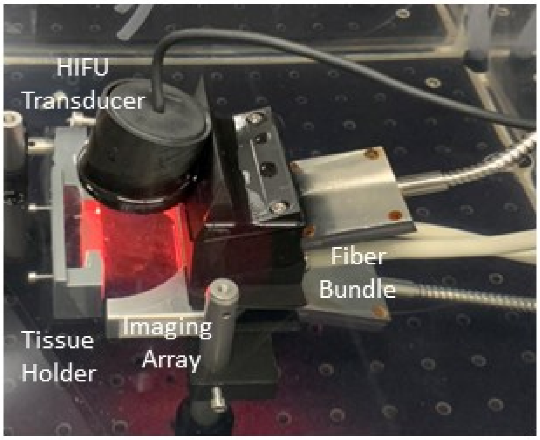

Supplementary Fig. S4. Experimental setup of the OPUS visualization of HIFU lesion progression in fixed *ex vivo* liver specimens.
